# Data on dissolved metals in Terengganu waters of South China Sea during pre-, post-, and Northeast Monsoon season

**DOI:** 10.1016/j.dib.2019.104806

**Published:** 2019-11-15

**Authors:** Marinah Mohd Ariffin, Ghazali Adiana, Joseph Bidai, Lee Siang Hing, Mohd Yusoff Nurulnadia, Meng Chuan Ong, Hasrizal Shaari, Siriporn Pradit

**Affiliations:** a Faculty of Science and Marine Environment, Universiti Malaysia Terengganu, 21300 Kuala Nerus, Terengganu, Malaysia; b Institute of Oceanography and Environment, Universiti Malaysia Terengganu, 21300 Kuala Nerus, Terengganu, Malaysia; c Marine and Coastal Resources Institute, Prince of Songkla University, 5th Floor, Academic Building Prince of Songkla University Hat Yai, Songkhla 90110, Thailand

**Keywords:** Dissolved metals, Northeast monsoon, Peninsular Malaysia coast, South China Sea

## Abstract

Metals are natural elements existed in the environment. However, due to the rapid development of urbanisation and economic, high content of anthropogenic metals are being perceived in polluting the environment. The oceans are known to be a part of the sinking basin for anthropogenic metals ends. Dataset provided is purposely to give an overview of dissolved metals spatial distribution in the South China Sea off the east Peninsular of Malaysia during the pre-, post- and Northeast (NE) Monsoon period. Seawater samples were collected in a grid of 18 stations at 3 different water depth. Dissolved metals were pre-concentrated on-board ship using Chelex-100 resin and analysed using Inductively Coupled Plasma Mass Spectrophotometry (ICPMS). The dataset shows the effect of NE Monsoon on dissolved metals spatial distribution mainly at the area closer to the land. Therefore, this dataset could reveal the past information on anthropogenic metals intrusion in the South China Sea, since Terengganu state was recently pointed to be one of the Malaysian waterfront city. Additionally, this dataset also could help in studying the cycle of metals in the southern South China Sea waters.

**Table:** Specifications Table

Subject	Oceanography, analytical chemistry
Specific subject area	Dissolved metals and Northeast monsoon effects
Type of data	Table
How data were acquired	Dissolved metals were pre-concentrated on-board during the sampling period using Chelex-100 resin and the concentration of metals was determined using Inductively Coupled Plasma Mass Spectrophotometry (ICP-MS) ELAN 9000.
Data format	Raw Analysed
Parameters for data collection	The concentration of 9 dissolved metals namely aluminium (Al), cadmium (Cd), chromium (Cr), copper (Cu), iron (Fe), manganese (Mn), nickel (Ni), lead (Pb) and zinc (Zn) was collected at 3 different water depth (surface water which was 0.5 m–1 m below the water surface, 10 m and bottom waters).
Description of data collection	Water samples were collected at 18 stations in a grid, at 3 different water layers, off the South Terengganu coastal. Dissolved metals were pre-concentrated on-board during the sampling period using Chelex-100 resin. Sampling was carried out during May 2007, September 2007 and November 2007 represent post-monsoon, pre-monsoon and Northeast monsoon period respectively.
Data source location	Universiti Malaysia Terengganu. Terengganu, Peninsular Malaysia, Malaysia. Latitude and longitude for each stations are presented in the article.
Data accessibility	Data are presented in the article.

**Table:** 

**Value of the Data** • The researcher can use these data as a baseline data for further investigation of the dissolved metals distribution in the East Coast of Peninsular Malaysia waters. • The present data could be served as a useful information on tracing the sources of natural or anthropogenic metals which are being flushed out into the Terengganu coast waters. • Data on dissolved metals in the Terengganu coastal may provide a better picture on the effect of Northeast monsoon on the distribution and movement of metals in the South China Sea water column. • Researchers and Terengganu's stakeholders may use these data for a general overview for further investigation on metals in South China Sea waters in order to monitor the intrusion of anthropogenic metals from land development towards developing Terengganu as a renowned waterfront city. • The present data could complete the environmental data gaps on metal cycle in the South China Sea since most past data on metals were focusing on sediment and organisms of the South China Sea

## 1 Data

Terengganu coast is located at the east region of Peninsular Malaysia with most of the main rivers flushed out toward the South China Sea. Seawater samples were collected at 18 stations off the south Terengganu coast. Coordinates of the sampling locations are shown in Table 1 below.

**Table 1:** Sampling station coordinate.

Station	Latitude	Longitude
T1	4° 45.000′ N	103° 26.200′ E
T2	4° 45.000′ N	103° 28.500′ E
T3	4° 45.000′ N	103° 34.000′ E
T4	4° 45.000′ N	103° 42.021 E
T5	4° 45.000′ N	103° 50.134′ E
T6	4° 45.000′ N	103° 58.247′ E
T7	4° 29.500′ N	103° 28.100′ E
T8	4° 29.500′ N	103° 30.500′ E
T9	4° 30.000′ N	103° 35.700′ E
T10	4° 30.000′ N	103° 43.810′ E
T11	4° 30.000′ N	103° 51.920′ E
T12	4° 30.000′ N	104° 0.031′ E
T13	4° 15.000′ N	103° 28.600′ E
T14	4° 15.000′ N	103° 31.000′ E
T15	4° 15.000′ N	103° 36.400′ E
T16	4° 15.000′ N	103° 44.512′ E
T17	4° 15.000′ N	103° 52.620′ E
T18	4° 15.000′ N	104° 0.728′ E

In order to assess the effect of Northeast Monsoon towards the distribution of dissolved metals in the South China Sea, sampling was carried out during May 2007 (represent the post-monsoon period), September 2007 (represent the pre-monsoon period) as well as November 2007 (represent the Northeast Monsoon period). Seawater samples were collected in a grid of 18 stations at three (3) different water layers namely surface water (1 m from the sea surface), middle layer (10 m from the sea surface) and bottom layer (1 m above the sea-bed surface). The concentration of dissolved metals determined during the sampling period was recorded in Table 2, Table 3, Table 4 below.

**Table 2:** The concentration of dissolved metals (μg/L) during May 2007.

Water depth	Station	Al	Cd	Cr	Cu	Fe	Mn	Ni	Pb	Zn
1 m (Surface)	T1	10.3	0.568	0.072	1.05	35.2	1.78	0.71	0.172	4.78
	T2	13.6	0.497	0.210	1.07	33.2	1.61	0.63	0.124	4.70
	T3	14.8	0.328	0.335	1.42	139	3.21	4.33	0.126	7.13
	T4	6.97	0.100	0.452	1.38	134	2.45	3.66	0.094	4.93
	T5	8.01	0.090	0.649	1.28	105	1.88	3.93	0.076	4.50
	T6	9.66	0.151	0.130	1.14	34.1	0.43	0.61	0.041	4.42
	T7	11.5	0.072	0.063	1.15	19.5	0.33	0.04	0.104	4.21
	T8	6.59	0.137	0.105	0.98	37.0	0.42	0.61	0.071	6.45
	T9	8.58	0.257	0.121	0.92	23.9	0.49	2.56	0.211	4.91
	T10	11.0	0.110	0.257	1.43	246	0.53	3.49	0.145	5.84
	T11	6.14	0.079	0.150	1.04	31.1	0.49	0.40	0.034	4.08
	T12	7.24	0.139	0.317	1.10	159	2.32	7.69	0.057	5.26
	T13	7.30	0.074	0.563	1.22	100	0.43	1.83	0.133	5.01
	T14	10.3	0.070	0.120	0.85	137	0.44	7.86	0.029	3.05
	T15	5.04	0.092	0.508	1.91	151	1.40	7.79	0.087	5.24
	T16	6.78	0.048	0.243	0.97	130	2.34	7.22	0.072	4.58
	T17	14.9	0.040	0.158	0.50	101	1.71	5.71	0.042	3.70
	T18	4.31	0.038	0.145	1.15	53.4	0.94	0.55	0.092	3.70
10 m (Middle)	T1	8.34	0.558	0.075	1.03	35.0	1.61	0.76	0.244	5.19
	T2	12.3	0.495	0.213	0.96	29.9	1.56	0.68	0.127	5.20
	T3	12.6	0.323	0.346	1.25	139	3.20	4.36	0.139	7.63
	T4	4.78	0.097	0.474	1.28	130	2.23	3.72	0.286	5.02
	T5	7.07	0.085	0.665	1.17	104	1.74	3.97	0.164	5.05
	T6	5.12	0.150	0.133	1.04	30.5	0.40	0.64	0.042	4.91
	T7	11.5	0.020	0.066	1.04	17.0	0.17	0.08	0.122	4.54
	T8	6.17	0.122	0.119	0.96	36.9	0.26	0.61	0.104	6.75
	T9	5.77	0.255	0.123	0.88	23.6	0.45	2.63	0.250	5.14
	T10	10.2	0.106	0.330	1.43	245	0.44	3.61	0.190	6.83
	T11	2.31	0.048	0.203	0.96	25.4	0.05	0.43	0.200	4.09
	T12	3.57	0.117	4.65	0.74	133	2.27	7.76	0.069	6.08
	T13	3.88	0.038	2.66	0.45	95.0	0.20	2.00	0.192	5.05
	T14	8.09	0.043	0.141	0.76	127	0.41	8.38	0.054	4.87
	T15	0.76	0.084	0.566	1.88	142	1.33	8.02	0.121	9.21
	T16	0.07	0.045	0.426	0.89	124	2.17	7.39	0.078	6.29
	T17	12.3	0.037	0.350	0.35	85.7	1.69	5.81	0.046	4.65
	T18	3.43	0.027	0.158	1.08	52.4	0.92	0.95	0.093	3.77
Bottom	T1	6.80	0.520	0.093	0.60	33.3	1.54	1.00	0.268	5.71
	T2	12.0	0.489	0.215	0.36	29.4	1.53	0.69	0.176	5.99
	T3	12.1	0.317	0.366	1.04	137.4	3.17	4.46	0.178	8.53
	T4	0.31	0.066	0.489	0.97	129.6	2.18	3.83	0.306	5.28
	T5	6.41	0.080	0.700	0.96	102.5	1.73	4.03	0.179	5.37
	T6	4.05	0.133	0.196	0.91	29.3	0.40	0.73	0.119	5.01
	T7	10.3	0.019	0.086	0.91	16.8	0.14	0.10	0.235	4.66
	T8	5.26	0.117	0.120	0.87	36.1	0.25	0.68	0.149	7.03
	T9	5.62	0.253	0.132	0.76	23.5	0.44	2.79	0.385	5.15
	T10	3.20	0.088	0.619	1.30	226.6	0.26	4.05	0.203	7.51
	T11	1.56	0.021	6.14	0.89	16.7	0.01	3.94	0.233	4.94
	T12	2.84	0.085	4.72	0.71	128.5	1.92	11.85	0.128	6.10
	T13	0.85	0.016	6.36	0.40	79.4	0.09	3.36	0.359	5.94
	T14	6.75	0.027	0.966	0.70	124.7	0.37	9.79	0.193	5.91
	T15	0.45	0.063	0.737	1.87	136.5	1.32	10.01	0.165	11.2
	T16	0.07	0.034	24.2	0.88	123.7	1.68	10.21	0.134	8.19
	T17	12.0	0.028	0.674	0.14	84.1	1.68	5.93	0.049	5.33
	T18	0.30	0.021	14.8	1.07	51.2	0.91	2.62	0.149	4.57

**Table 3:** The concentration of dissolved metals (μg/L) during September 2007.

Water depth	Station	Al	Cd	Cr	Cu	Fe	Mn	Ni	Pb	Zn
1 m (Surface)	T1	12.6	0.548	0.029	0.85	7.34	0.747	0.610	0.269	2.51
	T2	12.1	0.098	0.026	1.80	11.5	1.18	0.246	0.210	2.21
	T3	11.2	0.060	0.037	1.49	9.33	0.337	0.231	0.250	2.11
	T4	8.99	0.061	0.028	0.96	9.74	0.424	0.232	0.165	1.56
	T5	11.1	0.112	0.029	0.61	7.63	0.194	0.231	0.180	1.64
	T6	11.4	0.071	0.025	1.03	9.72	0.197	0.243	0.208	2.20
	T7	8.27	0.125	0.038	0.70	10.1	0.871	0.266	0.241	1.86
	T8	10.6	0.061	0.033	0.76	7.31	0.480	0.167	0.170	1.27
	T9	13.5	0.058	0.030	1.42	7.68	0.383	0.195	0.198	1.87
	T10	9.93	0.057	0.037	0.79	7.79	0.217	0.244	0.416	2.01
	T11	13.0	0.080	0.036	0.57	8.36	0.191	0.255	0.234	3.55
	T12	12.8	0.043	0.036	0.68	7.27	0.167	0.152	0.153	1.42
	T13	8.50	0.162	0.029	0.62	9.20	0.667	0.222	0.260	2.04
	T14	11.0	0.068	0.036	0.76	6.88	0.697	0.276	0.309	2.22
	T15	10.5	0.052	0.029	0.66	6.51	0.397	0.291	0.275	2.22
	T16	10.5	0.071	0.035	0.78	8.53	0.340	0.315	0.272	2.14
	T17	9.97	0.060	0.049	0.68	6.91	0.193	0.305	0.337	2.54
	T18	11.3	0.045	0.028	0.67	7.11	0.153	0.295	0.245	1.68
10 m (Middle)	T1	10.8	0.641	0.032	0.87	7.17	0.572	0.660	0.341	2.95
	T2	10.1	0.109	0.051	1.70	8.23	0.558	0.260	0.216	2.34
	T3	9.30	0.060	0.040	0.78	6.04	0.285	0.283	0.253	2.51
	T4	7.72	0.059	0.053	0.79	8.06	0.230	0.263	0.246	2.06
	T5	9.78	0.110	0.041	0.58	7.56	0.177	0.260	0.193	2.15
	T6	9.24	0.067	0.039	0.92	7.65	0.148	0.247	0.255	2.29
	T7	6.82	0.164	0.061	0.69	6.12	0.649	0.322	0.433	1.97
	T8	8.44	0.058	0.035	0.64	7.09	0.440	0.231	0.201	1.81
	T9	11.6	0.056	0.045	0.81	6.98	0.243	0.240	0.286	2.37
	T10	8.99	0.053	0.075	0.69	6.84	0.195	0.260	0.426	2.35
	T11	8.96	0.063	0.038	0.56	4.74	0.158	0.291	0.236	2.40
	T12	8.24	0.041	0.048	0.56	6.15	0.142	0.200	0.239	1.73
	T13	8.33	0.110	0.032	0.56	6.66	0.510	0.261	0.278	2.27
	T14	10.9	0.054	0.046	0.74	6.34	0.430	0.284	0.318	2.39
	T15	8.35	0.049	0.043	0.63	6.39	0.236	0.286	0.307	2.28
	T16	10.1	0.067	0.046	0.74	6.64	0.260	0.330	0.288	2.54
	T17	8.83	0.050	0.050	0.68	6.65	0.154	0.371	0.376	2.95
	T18	8.45	0.044	0.037	0.66	5.59	0.152	0.360	0.252	1.76
Bottom	T1	9.75	0.376	0.051	0.44	5.52	0.504	0.898	0.365	3.99
	T2	8.60	0.071	0.060	1.09	5.84	0.491	0.320	0.313	3.91
	T3	6.62	0.057	0.042	0.70	5.51	0.260	0.287	0.302	3.04
	T4	7.44	0.053	0.056	0.58	5.36	0.199	0.299	0.370	2.85
	T5	9.36	0.059	0.061	0.50	5.87	0.152	0.364	0.232	3.05
	T6	8.75	0.061	0.068	0.61	7.58	0.143	0.257	0.343	2.55
	T7	6.87	0.106	0.075	0.59	5.84	0.597	0.430	0.453	3.16
	T8	3.97	0.027	0.039	0.43	4.15	0.378	0.233	0.228	2.13
	T9	10.8	0.049	0.080	0.63	5.17	0.230	0.295	0.301	2.46
	T10	8.33	0.047	0.076	0.56	6.69	0.163	0.401	0.432	2.46
	T11	7.92	0.044	0.102	0.44	3.57	0.155	0.381	0.312	2.07
	T12	7.17	0.025	0.051	0.44	4.30	0.135	0.221	0.246	2.01
	T13	7.21	0.080	0.051	0.51	6.47	0.481	0.277	0.391	2.28
	T14	9.68	0.059	0.053	0.64	6.22	0.337	0.306	0.411	2.78
	T15	8.22	0.048	0.043	0.59	5.57	0.226	0.350	0.353	2.65
	T16	9.19	0.049	0.048	0.62	6.53	0.211	0.339	0.382	2.75
	T17	8.39	0.049	0.060	0.57	6.56	0.152	0.536	0.512	3.24
	T18	8.30	0.043	0.063	0.53	4.38	0.136	0.500	0.296	2.55

**Table 4:** The concentration of dissolved metals (μg/L) during November 2007.

Water depth		Station	Al	Cd	Cr	Cu	Fe	Mn	Ni	Pb	Zn
1 m (Surface)		T1	123	1.29	0.126	0.564	51.7	1.42	0.936	0.277	8.42
		T2	37.7	0.129	0.112	0.384	35.3	0.523	0.511	0.072	5.95
		T3	18.9	0.132	0.101	0.723	57.1	0.867	0.829	0.116	7.71
		T4	16.1	0.146	0.147	0.694	53.8	0.731	0.835	0.148	7.45
		T5	16.2	0.128	0.228	1.22	183	0.216	0.688	0.144	5.75
		T6	18.6	0.104	0.392	0.613	47.3	0.560	1.65	0.123	6.76
		T7	33.0	0.574	0.180	0.467	41.2	0.603	0.613	0.149	5.45
		T8	44.7	0.283	0.318	7.05	1223	28.3	0.785	0.093	5.44
		T9	17.2	0.082	0.125	0.390	42.1	0.586	0.743	0.122	8.48
		T10	25.3	0.091	0.275	0.368	31.6	0.310	0.638	0.158	8.86
		T11	20.5	0.088	0.242	0.452	46.1	0.589	0.696	0.109	7.31
		T12	15.6	0.090	0.247	0.377	29.3	0.180	0.609	0.104	6.89
		T13	25.5	0.218	0.101	0.529	50.1	0.825	0.461	0.062	2.33
		T14	23.4	0.108	0.185	0.771	111	2.317	0.528	0.098	7.46
		T15	15.2	0.091	0.140	0.373	29.6	0.272	0.614	0.093	7.50
		T16	16.2	0.091	0.102	0.410	29.9	0.282	0.604	0.130	7.96
		T17	22.5	0.093	0.224	0.504	45.4	0.588	0.598	0.088	7.02
		T18	23.8	0.110	0.411	0.748	79.0	1.35	0.643	0.096	9.35
10 m (Middle)		T1	63.0	1.02	0.197	0.480	50.9	0.848	1.38	0.326	9.85
		T2	30.0	0.098	0.186	0.362	29.7	0.431	0.628	0.116	6.94
		T3	12.0	0.109	0.137	0.359	29.7	0.236	0.861	0.129	7.92
		T4	12.3	0.111	0.201	0.588	27.9	0.223	0.909	0.313	7.46
		T5	15.7	0.102	0.475	0.442	35.1	4.012	0.966	0.162	6.30
		T6	14.9	0.096	4.726	0.481	42.2	0.509	2.81	0.136	7.58
		T7	29.5	0.175	0.190	0.376	31.3	0.445	0.789	0.157	7.95
		T8	17.8	0.082	2.419	0.393	28.6	0.615	1.46	0.152	5.71
		T9	15.0	0.079	1.326	0.356	33.3	0.351	1.26	0.168	8.44
		T10	20.8	0.079	0.296	0.307	26.4	0.279	1.08	0.182	9.05
		T11	13.8	0.085	0.705	0.373	30.6	0.225	0.922	0.152	9.13
		T12	13.0	0.087	0.305	0.317	28.3	0.111	0.594	0.138	7.69
		T13	25.1	0.150	0.238	0.377	28.4	0.652	0.624	0.181	6.30
		T14	21.3	0.080	0.368	0.345	33.9	0.377	0.787	0.104	8.41
		T15	14.3	0.087	0.146	0.298	27.8	0.251	0.720	0.094	9.22
Bottom		T16	15.3	0.080	0.295	0.406	29.5	0.269	0.738	0.133	7.89
		T17	22.3	0.088	1.031	0.442	31.3	0.276	1.00	0.148	7.97
		T18	17.6	0.085	0.424	0.505	31.6	0.162	0.811	0.097	10.2
		T1	56.2	1.08	3.830	0.416	32.0	0.623	2.60	0.316	12.8
		T2	16.6	0.070	0.475	0.283	20.9	0.259	1.07	0.130	7.62
		T3	11.0	0.078	8.504	0.331	24.2	0.229	4.37	0.259	9.89
		T4	11.6	0.088	6.139	0.337	23.6	0.187	4.99	0.346	8.31
		T5	11.1	0.085	43.5	0.398	23.7	0.138	22.0	0.163	8.15
		T6	14.2	0.075	4.791	0.466	26.5	0.156	3.10	0.194	7.60
		T7	26.5	0.121	2.717	0.316	29.2	0.272	2.14	0.174	8.73
		T8	16.1	0.055	310	0.337	27.6	0.447	157	0.319	11.7
		T9	13.7	0.067	4.247	0.345	27.5	0.241	2.67	0.259	9.33
		T10	19.5	0.076	1.121	0.271	25.7	0.239	1.20	0.322	9.29
		T11	13.7	0.080	6.076	0.363	29.0	0.183	2.91	0.165	10.2
		T12	12.8	0.075	0.476	0.316	27.2	0.109	0.765	0.182	8.08
		T13	8.88	0.123	6.223	0.163	16.7	0.157	3.45	0.353	8.28
		T14	13.5	0.062	24.2	0.329	21.5	0.219	11.7	0.159	10.0
		T15	12.0	0.079	0.310	0.293	27.1	0.250	0.840	0.131	11.1
		T16	12.2	0.074	0.619	0.379	27.8	0.256	0.909	0.137	8.80
		T17	18.8	0.072	4.739	0.498	26.2	0.163	2.66	0.212	8.65
		T18	14.3	0.080	15.1	0.339	24.8	0.113	7.47	0.153	10.6

Generally, the recorded data shows that influence of Northeast Monsoon on the distribution of dissolved metals in the South China Sea due to high concentration of metals were observed at stations closer to the land. A similar finding was found by Refs. [[1], [2], [3], [4], [5], [6]] as high load of metals was flushed out from the land-based during heavily rain period.

## 2 Experimental design, materials, and methods

Sampling was carried out during May 2007, September 2007 and November 2007 represent post-, pre- and Northeast Monsoon season. Seawater samples were collected at surface (1 m), 10 m and bottom waters in a grid of 18 stations. The sampling area covered approximately 3289 km^2^ with the nearest and furthest station was located approximately 0.9 km and 61 km from the Terengganu coastline respectively. Seawater samples were collected using Mercos water sampler and pre-concentrated at once, on-board ship under Class-100 portable laminar flow bench. All plastic-wares were previously clean using DECON and soak in 10% of nitric acid in order to remove the impurities. Dissolved metals were pre-concentrated using Chelex-100 resin (sodium form, mesh size of 100–200, powder form, Bio-Rad Laboratories), packed in acid-washed PTFE tubes, washed using ammonium acetate in order to remove saline matrix [6,7] and were eluted using 2 M nitric acid, adopted from William [8] and Bowles et al. [9]. Next, the pre-concentrated dissolved metals were collected into acid-washed centrifuge tubes and kept under 4 °C prior transportation to the laboratory. The concentration of dissolved metals was then analysed using Inductively Coupled Plasma Mass Spectrophotometry, Perkin ELMER ELAN 9000. The analytical procedures were validated against CASS-4 (Nearshore Seawater Reference Material for Trace Metals from National Research Council of Canada). Metal recovery results together with the detection limits are recorded in Table 5 below. Blanks were measured to check any metals contamination. The concentration of dissolved metals in per litre basis was calculated Equation (1) below:

(1) $$Metal\;concentration\;\left(\mu g/L\right)=\;\frac{X}{Pre-concentration\;factor}$$

Remarks:
X: Raw data reading by ICP-MS (μg/L)

$$\text{Pre}-\text{concentration factor}=\;\frac{\text{Volume of sample passed through the Chelex}-100\text{ column}}{\text{Final volume of pre}-\text{concentrated sample}}$$

**Table 5:** Analytical quality control data for dissolved metals using CASS-4.

Element	Dissolved detection limit^a^ (μg/L)	CASS-4 certified value (μg/L)	CASS-4 measured value (μg/L)	Recovery (%)
Cd	0.0013	0.026 ± 0.003	0.027 ± 0.011	105.31
Cr	0.082	0.144 ± 0.029	0.15 ± 0.02	107.44
Cu	0.0406	0.592 ± 0.055	0.578 ± 0.093	97.58
Fe	0.17	0.713 ± 0.058	0.785 ± 0.097	110.12
Mn	0.033	2.78 ± 0.19	2.58 ± 0.36	92.96
Ni	0.091	0.314 ± 0.03	0.362 ± 0.012	115.36
Pb	0.0007	0.0098 ± 0.0036	0.0108 ± 0.0039	110.27
Zn	0.069	0.381 ± 0.057	0.411 ± 0.075	107.91

Note: ^a^Detection limit calculated as three time of the standard deviation of the procedural blanks (n = 9).
